# Role of Phytotherapy in the Management of BPH: A Summary of the Literature

**DOI:** 10.3390/jcm12051899

**Published:** 2023-02-28

**Authors:** Vaki Antoniou, Vineet Gauhar, Sachin Modi, Bhaskar Kumar Somani

**Affiliations:** 1 Department of Urology, University Hospital Southampton, Southampton SO16 6YD, UK; 2 Department of Urology, Ng Teng Fong General Hospital, Singapore 609606, Singapore; 3 Department of Interventional Radiology, University Hospital Southampton, Southampton SO16 6YD, UK

**Keywords:** BPH, prostate, phytotherapy, LUTS

## Abstract

Benign prostatic hyperplasia (BPH) describes the non-malignant enlargement of the prostate. It is both common and growing in incidence. Treatment is multimodal, involving conservative, medical, and surgical interventions. This review aims to examine the evidence base for phytotherapies, specifically analyzing their role in treating lower urinary tract symptoms (LUTS) attributable to BPH. A literature search was completed, specifically looking for randomized control trials (RCTs) and systematic reviews involving phytotherapy treating BPH. Specific emphasis was placed on exploring substance origin, the proposed mechanism of action, evidence of efficacy, and the side-effect profile. Several phytotherapeutic agents were evaluated. These included serenoa repens, cucurbita pepo, and pygeum Africanum, among others. For most of the reviewed substances, only modest effectiveness was reported. Generally, though, all treatments were tolerated well with minimal side effects. None of the treatments discussed in this paper form part of the recommended treatment algorithm in either European or American guidelines. We, therefore, conclude that phytotherapies, in the treatment of LUTS attributable to BPH, do provide a convenient option for patients, with minimal side effects. At present, however, the evidence for the usage of phytotherapy in BPH is inconclusive, with some agents having more backing than others. This remains an expansive field of urology whereby there is still more research to be done.

## 1. Introduction

Benign prostatic hyperplasia (BPH) is a condition commonly encountered by the general urologist. Importantly so, it is also a growing area, with the global number of patients suffering symptoms attributed to BPH hitting 94 million in 2019 [1]. Many factors influence the prevalence of this condition, including age, race, fitness status, and medications [1].

BPH is the histologically confirmed, non-malignant enlargement of the prostate gland. It is characterized by an increase in both the number and volume of prostate epithelial and stromal cell types. The resulting enlargement of the prostate gland can restrict bladder outflow, contributing to storage, voiding, and post-micturition lower urinary tract symptoms (LUTS). These can become bothersome to patients and influence their quality of life. Management of BPH is multimodal, with conservative, medical, and surgical treatments available. Medical management of LUTS attributed to bladder outflow obstruction (BOO) includes alpha-1 antagonists, 5-alpha-reductase inhibitors, and PDE5 inhibitors.

Phytotherapy is an alternative medical therapy for symptomatic LUTS. Phytotherapies are defined as treatments using plants or substances attained from plants (Table 1). Their usage dates back to ancient times, with evidence from 15 BC in Egypt [2]. In modern times, their application for LUTS spans the planet. Numerous phytotherapies have been examined globally, with variable success in the treatment of LUTS for BPH. This paper aims to review the currently available phytotherapy options for urologists and patients alike. We examine both botanicals (substances attained from plants) and nutraceuticals (products derived from food sources). The specific focus was on evaluating the mechanism of action, origin, evidence of efficacy, and side-effect profile. The discussion will also be centered on the advantages and disadvantages of phytotherapy treatments compared with their medical counterparts. 

## 2. Materials and Methods

Google Scholar and PubMed searches were completed in November 2022 using the terms ‘benign prostatic hyperplasia’, ‘lower urinary tract symptoms’, ‘phytotherapy’, ‘saw palmetto’, ‘pygeum africanum’, ‘cucurbita pepo’, ‘urtica dioica’, ‘epilobium angustifolium’, ‘hypoxis hemerocallidea’, ‘pinus pinaster’, ‘solanum lycopersicum’, ‘roystonea regia’, ‘secale cereale’, ‘linum usitatissumum’ and ‘isoflavones’. Relevant studies were reviewed, and the full text was analyzed. In a non-systematic review, all large studies in the English language were included in this paper, and a summary of the literature is presented in this paper.

### 2.1. Serenoa Repens (Saw Palmetto)

By far, one of the most researched botanical phytotherapies is serenoa repens (Saw Palmetto), found in the berry of the American dwarf palm tree. A search of ‘saw palmetto BPH’ on Google Scholar yields 3470 results. Despite the wealth of global evidence on serenoa repens, there remains much debate over its efficacy, with the American Urological Association (AUA) updating their 2003 guidelines, which suggested that ‘modest’ efficacy could be obtained through the use of serenoa repens, to the current guidelines, which conclude ‘no effect’ from the phytotherapy [26]. Similarly, it should be noted that no phytotherapeutic agents are included in their medical therapy for the BPH algorithm. EAU guidelines summarize that, whilst there is evidence for serenoa repens, it is classified as ‘weak’, with recommendations that patients should be counseled about potentially modest symptomatic improvements [27]. A consensus statement by a panel of global urologists recently concluded that serenoa repens is both ‘*safe to use*’ and ‘*should be considered as a treatment option for men with mild to moderate BPH/LUTS as an alternative to watchful waiting*’ [28]. The most recent Cochrane review on serenoa repens included 32 randomized control trials (RCTs) and 5666 patients. This concluded that ‘*Serenoa repens, at double and triple doses, did not improve urinary flow measures or prostate size in men with lower urinary tract symptoms consistent with BPH*’ [29]. They also concluded that, compared to a placebo, serenoa repens did not decrease nocturia or prostate size, nor did it improve peak urinary flow [29].

Despite the controversy over potential efficacy, one thing that has been reiterated in the literature is that serenoa repens is well tolerated by patients [29]. Across the studies included in the Cochrane review, very few reported only minimal dropouts due to side effects [29]. Moreover, in the papers that compared serenoa repens to a placebo, there was no significant difference in drug-related adverse events [30]. The established mechanisms of action for serenoa repens include the inhibition of 5-alpha reductase, the inhibition of dihydrotestosterone receptor binding, and the reduction of inflammation and proliferation in the prostate [3,4,5]. Dosage options ranged from 450 mg to 3.6 gm.

### 2.2. Pygeum Africanum (Prunus Africana)

Pygeum Africanum is a supplement derived from the bark of the African prune tree (Rosaceae family). A 2002 Cochrane review, involving 1562 patients across 18 RCTs, concluded both symptomatic and flow-rate improvement (23% increase in peak flow) when compared to a placebo [31]. In this review, the dropout rate (13%) was similar to that of the placebo (11%) [31]. RCTs have also demonstrated improvement in both IPSS and uroflowmetry outcomes (peak flow increased between 10 and 35% across the treatment period) [32,33].

The mechanism of action in treating LUTS has been concluded from in vitro and in vivo studies on small mammals and has been reported to be two-fold, affecting both the prostate and bladder [34]. In the prostate, pygeum africanum inhibits growth factors (including EGF and f 5-lipoxygenase metabolites), has anti-androgenic effects, and causes apoptosis of stromal cells [6,7,8,9,35]. In the bladder, pygeum africanum induces a histamine-mediated protective response on detrusor contractility [10]. It must be said, however, for pygeum africanum that there is a lack of contemporary evidence for its efficacy. Also, studies thus far have only compared pygeum africanum to placebo medication rather than other established therapies.

Pygeum Africanum is available in tablet, powder, and capsule forms from numerous USA online retailers. Available dosages of pygeum africanum range from 100 mg to 4.5 gm per tablet/capsule. Interestingly, both efficacy and safety have been demonstrated in either one or divided 100 mg daily doses compared with a placebo [36]. There are also available combination tablets with serenoa repens.

### 2.3. Cucurbita Pepo

Cucurbita pepo is an oil derived from the common pumpkin seed. Its efficacy remains debatable in the literature. One paper from the 1990s concluded that, after 10 months of treatment with cucurbita pepo-based tablets, both subjective and objective (with uroflowmetry) benefits were evident [37]. A more recent small study from Iran showed post-treatment improvement in IPSS scores for men over 50 [38]. The same study, however, also concluded that tamsulosin led to a greater IPSS improvement at 1 and 3 months of treatment, albeit with a higher side-effect profile [38]. In fact, none of the patients in the cucurbita pepo group reported any side effects over the 3-month treatment period [38]. A similar result was found in a 2022 paper by Theil et al., who concluded that over 12 months, post-treatment scores improved for both IPSS (a 4.7 average reduction from baseline) and quality of life (QoL), with very minimal impact on sexual function (measured with the IIEF-5) [39]. Despite initial positive outcomes, a recent systematic review from Cuba concluded that there was ‘moderate’ evidence to suggest that cucurbita pepo did not clinically or functionally improve BPH in terms of prostate size or uroflowmetry measures [40]. Cucurbita pepo has also been investigated in its efficacy when used in combination with serenoa repens. Hong et al. concluded, in a trial including 47 men that, whilst not statistically significant, combination therapy appeared to have more of a symptomatic benefit than the individual counterparts (a 14.3 point reduction in the IPSS and 2.2 point improvement in QoL scores over 12 months in the combination group) [41].

The mechanism of action for cucurbita pepo is speculated to be multi-modal in nature. A blockage of dihydrotestosterone (DHT) binding has been demonstrated in human prostate cells for anti-androgen effects [11], and IFN-γ mediated, anti-inflammatory effects have been shown within in vitro studies [13]. Moreover, cucurbita pepo has been suggested to inhibit 5-alpha-reductase activity [12]. In addition to potential effects on prostate tissue, it has been suggested from rodent studies, that cucurbita pepo improves muscle relaxation through nitric oxide release [14]. Cucurbita pepo is available in both capsule and liquid oil forms.

### 2.4. Urtica Dioica

Urtica dioica is more colloquially known as the common stinging nettle—a plant often associated with the dermatitic rash it can cause when encountered. One double-blind RCT of 558 men demonstrated significant superiority of urtica dioica when evaluating the effect on IPSS scores (an 8-point reduction), post-void residual (37 mL reduction), and prostate size (4.8 cc reduction), compared to a placebo [42]. Following this, a meta-analysis from 2016 concluded that the usage of urtica dioica for the treatment of LUTS in BPH is both efficacious and safe [43]. In this meta-analysis, on average, IPSS scores were reduced by 18.1 and prostate volumes by 3.6 cc [43]. All included RCTs in this meta-analysis did not report any side effects or adverse events [43].

The mechanism of action of urtica dioica has been shown to involve 5-alpha-reductase inhibitory activity on testosterone [16]. Antiproliferative effects have also been demonstrated to affect just epithelial cells [15]. It is further speculated to inhibit the growth of the prostate in BPH [16].

### 2.5. Epilobium Angustifolium

Epilobium angustifolium represents an extract derived from a flower of the willowherb family. It should be noted that epolobium angustifolium has, thus far, been featured in very few human studies. One of these RCTs included 128 men; over 6 months, it was concluded that treatment with *E. angustifolium* extracts (EAEs) reduced both the number of patients reporting nocturia and also the number of patients with post-void residual scans (PVRs) of over 100 mL when compared with a placebo [44]. The same paper did not report any patient adverse events [44].

Its mechanism of action is thought to involve the down-regulation of androgens, anti-inflammatory medication, and the suppression of NF-κB [17]. Another study has implicated anti-proliferative effects through 5-alpha-reductase to also have an effect [18]. Epilobium angustifolium can be found in an oil drop form.

### 2.6. Hypoxis Hemerocallidea

This plant is endemic to southern Africa and is also known as the African star grass or African potato. Its main active component is β-sitosterol.

β-sitosterol is an extract that has been historically examined relatively meticulously. A 1997 double-blinded study of 177 patients found that 130 mg of β-sitosterol was significantly superior to placebo at 6 months with regards to the IPSS (mean 5.4 point improvement), Qmax (mean 4.5 mL/s improvement), and PVR (mean 33.5 mL improvement) [20]. Later, a Cochrane review was published in 1999 to analyze the clinical effect of these on symptomatic LUTS attributed to BPH [45]. A total of 519 men were included in this review across 4 RCTs [45]. B-sitosterols were found to improve both troublesome LUTS (an average of 4.9 IPSS points) and flow rates (an average peak flow improvement of 3.91 mL/s) [45]. Meanwhile, prostate size and withdrawal rates were comparable to the placebo group [45]. Gastrointestinal side effects were noted in 1.6% of these patients [45]. Unfortunately, there remains a lack of contemporary evidence for β-sitosterols. Serenoa repens, quercetin, and β-sitosterol have also been trialed in combination: A small-number, 3-month study demonstrated non-significant improvements in flow and PVR [46].

Hypoxis hemerocallidea has been shown to have anti-inflammatory, -neoplastic, -oxidant, -diabetic, and -infective properties in studies thus far [19]. Hypoxis hemerocallidea cannot be found as an isolated supplement.

### 2.7. Pinus Pinaster

Part of the Pinaceae family, pinus pinaster is colloquially known as the maritime pine, which can be found in the western Mediterranean. Its major active ingredient, similar to hypoxis, is β-sitosterol.

One Italian study examined 320 patients who were given a combination of 320 mg of serenoa repens, 120 mg of urtica dioica, and 5 mg of pinus pinaster, for a minimum of 30 days. Of the 80 patients who had evaluated post-treatment IPSS scores, 85% reported significant benefits, with minimal recorded side effects (a tiny proportion with gastrointestinal symptoms) [47]. However, it should be said that some of these patients also received congruent antibiotics or alpha-blockers. Moreover, no significant benefit was found from this combination in uroflowmetry studies or prostate volume measurements. Pinus pinaster can be found in tablet form.

### 2.8. Solanum Lycopersicum (Lycopersicum esculentum)

Solanum lycopersicum is a product of the common tomato. It is high in lycopene, which is a carotenoid antioxidant. In a review, lycopene supplementation was shown to decrease the incidence of both BPH and prostate cancer diagnoses, although this was not a statistically significant result [48].

One paper from Italy evaluated the impact of combination therapy from both phytotherapies as well as an alpha-blocker (alfuzosin) [49]. Serenoa repens, solanum lycopersicum, lycopene, and bromelain were all given in addition to alfuzosin as the treatment arm, with alfuzosin monotherapy being the control arm. The authors reported on 250 subjects and concluded that, at 6-month follow-up, combination therapy significantly improved IPSS scores (median 15-point improvement compared with 12 for the alfuzosin group), Qmax (median 16.7 mL/s vs. 13.8 mL/s), and PVRs (102 mL reduction compared with 109 mL) [49]. Of note, the number of patients reporting side effects was similar (16 in the combination group and 15 in the control group) [49]. Reported side effects in both groups included ejaculatory dysfunction, postural hypotension, and gastrointestinal upset [49].

Lycopene has a number of suggested mechanisms of action. To summarize, these include but are not limited to the inhibition of growth factors, apoptosis, and 5-alpha-reductase inhibition [21].

### 2.9. Roystonea Regia

Roystonea regia is a palm tree found in both North and South America. The oils from the harvested fruits (named D-004) are the active component used to treat BPH.

Its action has been shown, in both in vivo and in vitro studies, to provide antioxidant effects, inhibit 5-alpha-reductase, and reduce the contraction of the smooth muscle in the isolated deferens tube [22,23,50]. As of yet, clinical studies remain limited, although a promising comparative study of 100 men over 6 months demonstrated the superiority of roystonea regia over an alpha-blocker (terazosin) in IPSS scores (a 9.5-point improvement versus 8.4 points) and a reduction in prostate size (3.4 cc versus 1.4 cc) [51]. In the same study, both groups improved PVR, whilst roystonea regia was better tolerated [51]. One patient in the roystonea regia group developed urinary sepsis over the 6-month period [51]. A further comparative study of roystonea regia and serenoa repens concluded that they were equal in their efficacy of improving LUTS from BPH [51]. Roystonea regia is not available online as an isolated supplement.

### 2.10. Secale Cereale

This is a plant belonging to the Graminaceae family, commonly known as rye. Therapy has been established through the use of extracts from the pollen of the plant.

A Cochrane review completed in the late 1990s concluded, from studies involving a total of 440 patients, that it is well tolerated and modestly improves overall urologic symptoms including nocturia [52]. However, they also stated that evidence was limited by a number of factors, including gaps, short duration, limited subject numbers, and the unregulated quality of preparations (with a lack of dosages provided in all cases) [52]. A further literature review two years later concluded the same outcome [53]. In this review, the authors concluded that whilst Cernilton (containing rye pollen extract) improved nocturia (−0.4 times per night, on average), there were no significant differences in terms of uroflowmetry, PVR, or prostate volume [53]. Since these studies at the turn of the millennium, there have been two further studies of note. The first is a comparative study looking at therapeutic groups containing an alpha blocker, 5-alpha-reductase inhibitor, or cernilton [54]. The authors concluded that each treatment subjectively and objectively improved LUTS from BPH, with no difference between the treatment arms over 15 months [54]. The second compared bi-daily doses of secale cereale (375 mg vs. 750 mg), concluding that the higher dose was more efficacious without any reported adverse effects [55]. Despite these early studies, it should be noted that there has been a lack of evidence on secale cereale in the last 15 years.

The mechanism of action of secale cereale Is unknown, although many different modes have been suggested, including the relaxation of the urethral sphincter smooth muscle, apoptosis in prostate epithelial cells, the blockage of alpha adrenoceptors, and the inhibition of 5-alpha-reductase [21]. Secale cereale supplementation is available in tablet form.

### 2.11. Linum Usitatissimum

Linum usitatissimum is more commonly known as flax or linseed. Typically, its usage is predominated by the textile industry, where it is used to make various linen-based products, such as bed sheets. Initial studies in rats demonstrated the efficacy of a diet enriched with flax on prostate size and the prostate-to-weight ratio [24]. Clinical studies have also shown some benefits. A small group of patients who had a flaxseed-supplemented diet for 6 months showed some decrease in prostate epithelium on biopsy [56]. A further double-blinded RCT demonstrated efficacy, over 4 months at different doses of a flaxseed extract, on IPSS (a 6.88-point reduction in the 600 mg group) and quality of life scores (a 1.75-point improvement in the 600 mg group) [57]. The major theoretical concern regarding high doses of linum usitatissimum is that it contains around 20–50 mg of cyanide per 100 g [58].

To date, there have been three proposed mechanisms of action for linum usitatissimum—anti-inflammatory, 5-reductase inhibition, and the inhibition of growth through growth factor modification [58]. Flaxseed supplements are widely available in a number of forms and doses. 

### 2.12. Isoflavones

Isoflavones could also have a role in BPH management. Isoflavones are found in soy-rich foods and have previously been suggested to improve BPH due to oestrogen receptor, activation-mediated apoptosis [25]. This link was suggested due to the concurrently high soy consumption and low prostate cancer rates in some Asian countries. Evidence was provided for this theory in initial rat studies, which showed reductions in the size of the prostate in the isoflavones-treated, BPH-induced mammals [59]. An Italian meta-analysis of PSA in human patients treated with isoflavones, however, refuted this idea [60].

### 2.13. Others

Other research has focused on lifestyle modifications, which could improve LUTS associated with BPH. In particular, the eastern diet, rich in vegetables (β-carotene, vitamin C, and vitamin E), fish (eicosapentaenoic acid and docosahexaenoic acid), tomatoes (lycopene), and soy (isoflavones), have all been discussed [61]. Moreover, the positive effect of elocalcitol, a vitamin D receptor agonist, on slowing prostate growth [62] has led to vitamin D being discussed as a potential supplement that could help symptoms.

Finally, there are other phytotherapies/nutraceuticals that have shown positive preliminary findings in research. Opuntia ficus-indica (prickly pear) is one that has been suggested to improve urgency associated with BPH; its mechanism of action is yet unknown [63]. Another is telfairia occidentalis, which is a leafy vegetable, part of the Cucurbitaceae family, and grown in West Africa. Whilst initial outcomes have shown that treatment could reduce the size of the prostate, the mechanism of action is unknown and research has been limited to small-number rat studies [64,65].

## 3. Discussion

The following review has provided a summary of the agents available to patients for the treatment of their LUTS. Phytotherapies have enjoyed widespread sales globally, partly from the availability and popularity of health food stores. Old data from 1994 has shown annual US sales from these stores to gross $553 million [66], and sales over the past 3 decades are sure to have increased further. In fact, as evidence of this point, in the early millennium, 1 in 2 people used at least one dietary supplement regularly, with over two-thirds of those over the age of 70 using them [67].

It must be said that phytotherapies confer certain advantages for patients. Firstly, the ability to purchase them online or over the counter, without a prescription, represents ease of access. This is especially important in the post-pandemic era when accessing a primary care doctor is harder than ever. Moreover, as conferred by human studies for these agents, many phytotherapy treatments are associated with low side effect profiles.

Having said that, phytotherapies should be used with an element of caution. In all cases described above, the mechanism of action is not completely known or understood. This, of course, is not a prerequisite to medical treatment, though. Historically, many medications have been used for treatment before a complete understanding of their mechanism of action has been developed. Further to this point, whilst phytotherapy usage has spanned centuries, many of these substances have not gone through the rigorous testing that medication would normally have undergone. This is partly reflected in their lack of recommendation in the European or American guidelines. For all therapies, the most important aspect is their efficacy. For most of the phytotherapies, the literature has shown modest effects, at best. Often, when compared to established medications, phytotherapy has been shown to be inferior.

Another difficulty of phytotherapy conferred on patients is that the dosage of these therapies can prove difficult to manage. Caution should be placed on in vitro studies, as some of these studies use extremely high levels of the ingredient in relation to the cells examined. These dosages may be far above the therapeutic level in humans. Additionally, the active ingredient for many of these substances remains unknown. This can muddy the water when considering the dosage to be delivered or comparisons between different options. Moreover, it can lead to confusion for patients buying these substances over the counter. The point stands, though, that many of these agents have a very minimal side effect profile, which is encouraging.

All of these substances are found in common plants or vegetables. However, there is minimal research comparing taking a supplement to the inclusion of the active fruit or herb into the diet. A lot of these substances, when ingested as part of food, obviously have other additional benefits, including nutritional values, vitamins, and antioxidants. This is an area of future research that is yet to have been undertaken for many of the phytotherapeutic agents. Moreover, long-term comparative studies between phytotherapy, medical management, and new, minimally invasive surgical treatments for BPH would also help in patient counseling and informed decision-making about treatment options [63,64].

In a detailed review of the phytotherapy options available to both patients and surgeons, there is no clear standout agent. AUA guidelines state that current evidence on phytotherapies suffers from ‘*multiple shortcomings*’ [26] (including single centers, a lack of placebo comparison, and a lack of intention-to-treat analysis), whilst EAU guidelines similarly state that ‘*heterogeneity and a limited regulatory framework*’ affect conclusions [27]. Certainly, the option with the most evidence base is serenoa repens. It represents the only one out of the current options that is mentioned in both the AUA and EAU guidelines. Even this agent, though, comes under strict scrutiny, given that two independently conducted, double-blinded trials comparing serenoa repens to a placebo found no clinical superiority [26]. From the research presented in this paper, it is difficult to accurately conclude which would be the best agent for patients, and conversely, whether any agents should be avoided. Certainly, most of the papers have concluded that various phytotherapy agents are safe and come with a minimal side effect profile. At present, the agents with the least in vivo evidence base include isoflavones, epilobium angustifolium, and pinus pinaster. While these therapies could be explored more intensively, it is possible that it does not happen more often because the active content may vary according to the plant genetics, the quality of soil, climatic factors, the extraction method, and the time of harvesting. Moreover, they are often poorly controlled and standardized. 

This paper has aimed to provide a summary of the data available on phytotherapies in the treatment of LUTS attributable to BPH [30,36,37,38,68,69,70,71,72] (Table 2). The present study has used select studies to decipher, where possible, the main conclusions from existing evidence for the use of these substances. Modern studies, where available, have been added to provide a contemporary update from previous reviews. Perhaps with the advent of new, minimally invasive surgical therapies (MIST) for BPH [73,74,75], further randomized trials between phytotherapy and MIST need to be conducted, given that some of these therapies are for smaller prostate sizes [73,74] and phytotherapy can potentially compete with these. 

## 4. Conclusions

Our review has provided a contemporary overview of potential phytotherapy treatment options for patients worldwide. It is clear to see that, at present, evidence for phytotherapy is inconclusive, with some agents having more evidence base than others. Perhaps the most important message to be taken away for patients considering phytotherapy as a treatment option is that, prior to any self-medication, appreciation must be placed on the fact that benefits may be limited. Conversely, the main positives, currently, to phytotherapies are their encouraging side effect profile and ability to be attained with relative ease and without prescription. 

## Figures and Tables

**Table 1:** Provides an abbreviated summary of phytotherapies for LUTS.

Name	Origin	Proposed Mechanisms of Action for BPH
*Serenoa repens (Saw Palmetto)*	Fruit from a dwarf palm tree	Non-competitive inhibition of 5-alpha-reductase [3] Inhibition of androgen receptor dihydrotestosterone binding [4] Reduction of prostate inflammation [5]
*Pygeum Africanum (Prunus Africana)*	Bark from an evergreen tree, part of the Rosacea family	Anti-androgen by inhibiting androgen and progesterone receptors [6] Inhibition of prostate cell proliferation and apoptosis of stromal cells [7,8] Anti-inflammatory through inhibition of 5-lipoxygenase [9] In the bladder, histamine-mediated protective response to the detrusor muscle [10]
*Cucurbita pepo*	Oil from pumpkin seeds	Competitively inhibit dihydrotestosterone binding [11] Inhibition of 5-alpha-reductase [12] IFN-γ-mediated anti-inflammatory effects [13] Muscle relaxation through nitric oxide release [14]
*Urtica dioica*	Roots of the common stinging nettle	Anti-inflammatory and anti-proliferative [15] Inhibition of 5-alpha-reductase [16] Inhibition of prostate growth [16] The leaves of this plant also contain β-sitosterol, which inhibits prostate cell proliferation
*Epilobium angustifolium*	Aerial parts of the flowering willow herb	Down-regulation of androgens [17] Anti-inflammatory and anti-proliferative [17] Inhibition of 5-alpha-reductase [18]
*Hypoxis hemerocallidea*	Tuber (underground part of the stem) of the African star grass (African potato)	Anti-inflammatory activity, inhibiting COX-1 and COX-2 activity [19] Contains β-sitosterol, which inhibits prostate cell proliferation [19,20]
*Pinus pinaster*	Twigs and resin from the maritime pine, part of the Pinaceae family	Contains β-sitosterol, which inhibits prostate cell proliferation [20]
*Solanum lycopersicum (Lycopersicum esculentum)*	Tomatoes (Lycopene, also found in watermelon, peaches, and red berries)	Contains lycopene, which is the active substance Inhibition of 5-alpha-reductase [21] Reduction of prostate growth by inhibition of growth factors [21] Inducing apoptosis of prostate cells [21]
*Roystonea regia*	Mature fruit of a palm tree	Inhibition of 5-alpha-reductase [22] Reduced contraction of smooth muscle in the deferens tube [23]
*Secale cereale*	Pollen from the rye plant of the Graminaceae family	Inhibition of 5-alpha-reductase [21] Relaxation of urethral sphincter tone [21] Apoptosis of prostate epithelial cells [21] Block of alpha-adrenergic receptors [21]
*Linum usitatissimum*	Oil from the common flax	Anti-inflammatory [24] Inhibition of 5-alpha-reductase [24] Inhibition of growth through growth factor modification [24]
*Isoflavones*	Soy products	Oestrogen receptor activation-mediated apoptosis [25]

**Table 2:** Summary of relevant literature examining the role of phytotherapies in treating LUTS attributable to BPH.

Name	Authors	Number of Participants	Country of Origin	Comparison Arm	Follow-Up Period	Summary of Results
*Serenoa repens (Saw Palmetto)*	Bent et al., 2006 [68]	225	United States	Placebo	14 months	No significant reduction in prostate volume (mean difference −1.22 cc), peak flow (mean difference 0.43 mL/s), or residual volume (mean difference −4.51 mL), after treatment with Serenoa repens compared with placebo.
*Serenoa repens (Saw Palmetto)*	Barry et al., 2011 [30]	369	United States	Placebo	72 weeks	No significant difference in American Urological Association Symptom Index (AUASI) scores after treatment with Serenoa repens compared with placebo (mean difference −0.79 points).
*Serenoa repens (Saw Palmetto)*	Hizli & Uygur 2007 [69]	60	Turkey	Tamsulosin	6 months	No significant improvement in peak flow (mean difference −0.4 mL/s), International Prostate Symptom Scores (IPSS) (mean difference 1.5 points), prostate volume (mean difference −0.3 cc), or residual volume (mean difference 4.6 mL), after treatment with Serenoa repens compared with tamsulosin (mean difference −0.4 mL/s).
*Pygeum africanum (Prunus Africana)*	Barlet et al., 1990 [70]	263	Germany, France, and Austria	Placebo	60 days	Improvement in peak flow rate after treatment with pygeum africanum compared with placebo (mean difference 1.1 mL/s). No significant improvement in residual volume rate after treatment with pygeum africanum compared with placebo (mean difference -12.6 mL).
*Pygeum africanum (Prunus Africana)*	Chatelain et al., 1999 [36]	209	France	None	12 months	Single or bi-daily dosing had similar efficacy. IPSS scores improved from baseline by between 35 and 38%. Qmax increased by between 1.63 and 2.02 mL/s.
*Cucurbita pepo*	Theil et al., 2022 [39]	130	Germany	None	24 months	IPSS was improved at 12 months, on average, by 4.7 points. IIEF-5 scores indicated a minimal impact on sexual function.
*Cucurbita pepo*	Zerafatjou et al., 2021 [38]	73	Iran	Tamsulosin	3 months	No significant difference in International Prostate Symptom Scores (IPSS) after treatment with cucurbita pepo compared with tamsulosin (mean difference 1.81 points). No significant difference in prostate volume after treatment with cucurbita pepo compared with tamsulosin (mean difference 0.72 cc). No significant difference in peak flow after treatment with cucurbita pepo compared with tamsulosin (mean difference 1.71 mL/s).
*Urtica dioica*	Safarinejad 2005 [42]	558	Iran	Placebo	6 months	Significant improvement in peak flow (mean difference 4.8 mL/s), IPSS (mean difference 6.5 points), and residual volume (mean difference 37 mLs), after treatment with urtica dioica, compared with placebo.
*Urtica dioica*	Hosseinabadi et al., 2014 [71]	248	Iran	Prazosin	2 months	3 g/5 g/7 g, in combination, significantly improved IPSS scores after treatment, compared to treatment with prazosin alone (mean change in IPSS 10.46 points in the 7 g urtica dioica group compared with 2 points in the control group)
*Epilobium angustifolium*	Esposito et al., 2021 [44]	128	Italy	Placebo	6 months	Epilobium angustifolium was significantly more effective than placebo for IPSS scores (mean difference 2.5 points) and residual volume (mean difference 4.3 mL), but not for prostate volume (mean difference −1.3 cc).
*Hypoxis hemerocallidea*	Berges et al., 1995 [72]	200	Germany	Placebo	6 months	Peak flow (mean difference 4.1 mL/s), IPSS placebo (mean difference 5.3 points), and residual volume (mean difference 23.8 mL) were improved more in β-sitosterol (the active ingredient in hypoxis hemerocallidea) than placebo (mean difference 4.1 mL/s). There was no difference between prostate volume after treatment with β-sitosterol compared with placebo.
*Hypoxis hemerocallidea*	Klippel et al., 1997 [20]	177	Germany	Placebo	6 months	Hypoxis hermerocallidea was significantly more effective than placebo for IPSS scores (mean difference 5.4 points), residual volume (mean difference 33.5 mL), and peak flow (mean difference 4.5 mL/s).
*Pinus pinaster*	Pavone et al., 2010 [47]	80	Italy	None	12 months	Combination therapy (serenoa repens 320 mg, urtica dioica 120 mg, and pinus pinaster 5 mg)—85% of patients reported symptomatic improvement in LUTS. No improvement in prostate size or peak volume.
*Solanum lycopersicum (Lycopersicum esculentum)*	Lambertini et al., 2021 [49]	250	Italy	Alfuzosin	12 months	Combination therapy (alfuzosin, serenoa repens, solanum lycopersicum, lycopene, and bromelain) compared with alfuzosin alone. Combination therapy significantly decreased IPSS compared to the control group (mean difference of 5 points) and residual volume compared with the control (mean difference of 23 mL).
*Roystonea regia*	Guzmán et al., 2019 [51]	100	Cuba	Terazosin	6 months	D-004 (containing roystonea regia) was more effective than terazosin in reducing the IPSS score (mean difference of 1.1 points). There was no significant difference between the two groups with regard to effects on prostate volume and residual volume.
*Secale cereale*	Xu et al., 2008 [55]	240	China	Lower dose of secale cereale (375mg)	4 years	Cernilton (containing secale cereale), administered at 750 mg daily, was more efficacious than 375 mg daily at reducing IPSS (mean difference 5.3 points), residual volume (mean difference 11.1 mL), and peak flow (mean difference 9.5 mL/s)
*Linum usitatissimum*	Zhang et al., 2008 [57]	78	China	Placebo	4 months	Linum usitatissimum did not show significantly improved IPSS or peak flow over placebo. Despite this, treatment for 4 months with 600 mg of linum usitatissimum reduced IPSS by 6.88 points and improved peak flow by 2.7 mL/s.

## Data Availability

Available and can be provided on request.
